# Prevalence of Bullying Behaviors Among Students From a National University in the United Arab Emirates: A Cross-Sectional Study

**DOI:** 10.3389/fpsyg.2022.768305

**Published:** 2022-04-25

**Authors:** Fatima Al-Darmaki, Haleama Al Sabbah, Dalia Haroun

**Affiliations:** ^1^Department of Psychology, College of Natural and Health Sciences, Zayed University, Abu Dhabi, United Arab Emirates; ^2^Department of Health Sciences, College of Natural and Health Sciences, Zayed University, Dubai, United Arab Emirates

**Keywords:** bullying behavior, anxiety, depression, aggression, PTSD

## Abstract

**Background:**

This study aims to investigate bullying behaviors among college students at one of the national universities in UAE, and also to examine the psychological characteristics of those who were exposed to, or have experienced bullying.

**Methods:**

A cross-sectional study was conducted on 839 undergraduate students at one of the national universities in the UAE. Students from all colleges participated in this study and were selected by using stratified random sampling. Participants completed a bullying survey designed for the study, in addition to three psychological measures [i.e., Aggression Questionnaire, Buss and Perry, 1992; The Primary Care Anxiety and Depression, El-Rufaie et al., 1997; and the Post Traumatic Stress Disorder (PTSD) for Diagnostic and Statistical Manual of Mental Disorders-Fifth Edition (PCL-5), Weathers et al., 2013].

**Results:**

The prevalence rate of students being exposed to or engaged in bullying was 26.3% (221 out of 839). Of those, 72 students (8.7%) reported being bullied, 29 (3.6%) reported bullying others, and 185 (22.8%) reported witnessing friends being bullied. The most common types of bullying reported were traditional bullying (e.g., face-to-face bullying, verbal, and physical). Cyberbullying was not very common. More females reported being bullied in comparison to males and most of the aggressors were peer students. Overall, moderate level of aggressive personality traits and low levels of symptoms of depression, anxiety, and PTSD were reported for the total sample. *T*-tests revealed significant differences in the three psychological measures between those who did not experience bullying and those who did. The mean scores on the Aggression Questionnaire for those who bullied others were significantly higher than those who did not experience bullying.

**Conclusion:**

Experiences of bullying seem to impact college students’ mental health in the UAE. Therefore, efforts need to focus on developing preventive programs to increase students’ awareness of bullying and its negative impact on campus environment. Offering psychological help for those who were exposed to bullying would help them to deal effectively with this trauma.

## Introduction

Bullying is an intentional aggressive behavior that is carried out repeatedly, which usually occur between perpetrators and victims who are unequal in power. Factors like physical size, social status seem to empower aggressors to victimize other individuals (Nansel et al., 2004). Traditional face-to-face bullying is a form of aggression which can be verbal (e.g., name calling, threatening, blackmailing, or making derogatory comments), or physical (e.g., hitting, pushing around, or physical intimidation). It may also be indirect or relational, such as excluding victims socially, or spreading rumors (Carlyle and Steinman, 2007; Liang et al., 2007; Lund and Ross, 2017). A new form of bullying has emerged in the 2000s as an extension to traditional bullying; this occurs through electronic technologies which spreads bullying beyond school premises. This cyberbullying power is rooted from expertise on social media, rather than physical strength or social status (Hinduja and Patchin, 2008; Wachs et al., 2020).

Bullying can be further differentiated by type, but regardless of the label, research has proven that it has negative physical and emotional effects, and has a social impact on those who are involved in bullying as well as on others (e.g., Gruber and Fineran, 2008; Schenk and Fremouw, 2012; AlMulhim et al., 2018). Gender differences have been noted; males tend to bully and get bullied more than females, boys and younger students are more prone to take the aggressor’s side compared with girls and older students (Bjärehed et al., 2020). The type of bullying in which males are involved in is often of the direct traditional type, while females tend to be more involved in indirect/relational or manipulative forms of bullying (Hinduja and Patchin, 2008; Olweus and Limber, 2010; Lee, 2017). Despite this, both genders feel equally victimized (Chapell et al., 2004). As most research on bullying has been done internationally (e.g., Bjärehed et al., 2020; Wachs et al., 2021), we know very little about bullying in the United Arab Emirates (UAE). Therefore, the current study attempts to fill this gap by investigating experiences of bullying from a sample gathered at a UAE-based university.

Research indicates that bullying declines with age (Pepler et al., 2008) dropping from 15% in 2nd grade to 5% in 9th grade (Olweus, 1994). A large number of studies found bullying to peak during adolescence, then victimization gradually decreases with age (e.g., Pepler et al., 2008; Craig et al., 2009; Bjärehed et al., 2020). Longitudinal studies have demonstrated a continuum where being a bully/victim in elementary school is associated with continuing to be a bully/victim at high school and college (Sourander et al., 2000; Schäfer and Korn, 2004). Data from the WHO, Global School-based Student Health Survey (GSHS), and Health Behavior in School-aged Children (HBSC) on bullying prevalence rates in different regions revealed that the prevalence rates of bullying in the Middle East and North Africa were 41.1% and 42.7%, respectively, and the rate was 48.2% for Sub-Saharan Africa. A simple comparison between these rates and the rates of North America (31.7%), Central America (22.8%), and South America (30.2%) shows the difference. Data collected between 2002 and 2017 also revealed changes in bullying rates over time. For example, the prevalence rate of 35 out of 72 countries surveyed has increased, and 31 countries showed a decrease in bullying, whereas 24 showed no change (United Nations Education and Scientific Cultural Organization, 2019). Despite these informative results, most of these studies were conducted on children and adolescents (e.g., Wachs et al., 2019, 2021; Bjärehed et al., 2020).

Relevant to college students, Tanrikulu and Erdur-Baker (2019) surveyed bullying among Turkish university students. They found approximately half of the participants admitted to having cyberbullied someone two or more times during the past 6 months. Males had a significantly higher rate of cyberbullying compared to females.

The rates of bullying among university students were similar to that of high school students. In a review of 14 studies from 2004 to 2013 covering populations ranging from 119 to 2085 college students, Lund and Ross (2017) reported a general prevalence rate of bullying that ranges between 20% and 25%. Students who were bullied reported being victimized in traditional face-to-face bullying, such as verbal aggression, while 10%–15% reported being cyber-victimized. Furthermore, 20% of students reported bullying their peers in traditional non-cyber ways, while 5% cyberbullied their peers. A similar pattern has been reported in the literature where both genders felt victimized by the negative effects of bullying on their psychological and physical health (Chapell et al., 2004). Moreover, studies on bullying among teacher/professor-bully show that students have been bullied by their educators (Al-Hussain et al., 2008).

A link between bullying and aggressive behaviors and personality traits has been previously documented in the literature (e.g., Sigurdson et al., 2014; Rodkin et al., 2015; Pallesen et al., 2017). Aggressive behavior has been observed among university students and the stress involved in this transitional period was shown to increase their aggressive behavior (Lundskow, 2013). University students, especially males, who live in dorms were found to have a low tolerance threshold against stressful conditions and higher aggression rates compared to the students who live at home (Alami et al., 2015). Students who bullied others were found to have higher levels of aggressive behaviors than those who were not involved (Undheim and Sund, 2010) and to have low scores on agreeableness and conscientiousness personality dimensions than the victims or the control groups. Those who were bullied scored low on extroversion and neuroticism (Pallesen et al., 2017).

Previous research found that those involved in bullying reported greater symptoms of anxiety, depression, and post-traumatic stress disorder (e.g., Haynie et al., 2001; Nansel et al., 2004; Arseneault et al., 2010; Undheim and Sund, 2010) compared with those who did not experience bullying. In one study, post-traumatic stress disorder, depressive symptoms, and suicide were found to correlate significantly with cyberbullying and physical peer violence in youths who visited an urban emergency department (Ranney et al., 2016). Additionally, those who experienced pre-college bullying were more likely to report depressive and anxiety symptoms as well as a lower perception of mental and physical wellbeing than their non-bullied peers (Erdur-Baker, 2009; Klomek et al., 2011; Chen and Huang, 2015; Giovazolias and Malikiosi-Loizos, 2015). These findings were based on samples mainly from Western countries. The impact of bullying on the wellbeing of individuals from the UAE has not been documented. Relevant to this study, however, AlMulhim et al. (2018) studied 400 college students in Saudi Arabia and found that 49% of the population surveyed have experienced bullying by their peers previously during their school time. They also expressed high levels of anxiety and depression even during college studies long after they were bullied. Some researchers argue, however, that pre-college exposure to bullying does not necessarily mean that students will also be involved in bullying later as college students. Some can be resilient and can adjust well in their new college environment with new social experiences (Holt et al., 2014; Chen and Huang, 2015).

As bullying is known to exist worldwide in educational settings, its prevalence in the educational settings in the UAE has not been documented. To the best of our knowledge, there are no published data on bullying among university students in the UAE. This may be because such cases are rarely reported or documented. Although universities have student misconduct policies and disciplinary procedures, our observations as well as the observation of the administration of our institution indicate that student bullying is still occurring on campuses, creating fear and stress among students and their parents. Therefore, this study was carried out to address the size of the problem among the university students in order to design the appropriate interventions.

Interestingly the word “bullying” does not exist in the Arabic language (Kazarian and Ammar, 2013); only a translation “Tanamor” or in Arabic “تنمر” is used to refer to such cases. “Tanamor” implies power and aggressiveness toward those who are perceived as weak or lack power. In an Arab subculture, such as the Emirati culture, victims of bullying in schools or universities regarded it as an embarrassing incident; hence, it remains mostly unreported. In addition, parents may encourage their children to respond with violence, thus, making it even more problematic. However, recent efforts (e.g., social medial articles, bullying prevention initiatives, and school counseling outreach programs) to raise awareness attracted researchers’ attention to address the issue. Our observations indicate that there is a change in the mindset of student populations about bullying, and many students seem to be willing to report it to their families, friends, counselors, or administrators.

Moreover, the consequences of bullying on students’ mental health are unknown. Therefore, examining bullying behavior and its impact on students’ mental health in a sample of college students in the UAE would reveal interesting results. Such findings will help decision-makers and educators as well as counselors to develop interventions to tackle this problem.

The main purpose of this study was of two-fold: (a) to investigate the bullying behavior among a sample of college students from a national university in the UAE and (b) to examine the psychological characteristics of those who were exposed to or have experienced bullying. This research examined (a) prevalence rate of bullying and victimization, (b) types of bullying and the identity of perpetrators of bullying, (c) reasons for bullying, and (d) participants’ suggested strategies to deal with bullying on campus. Additionally, the impact of bullying on the victims’ psychological wellbeing was investigated. This was done through examining participants’ experiences with anxiety and depression (as measured by the Primary Care Anxiety and Depression Scale; El-Rufaie et al., 1997), symptoms of post-traumatic stress disorder (as measured by Posttraumatic Stress Disorder Checklist for DSM-5; Weathers et al., 2013), and personality traits of aggression (as measured by Aggression Questionnaire; Buss and Perry, 1992). Whether there were significant differences on these measures based on the different types of bullying behaviors (i.e., being bullied, being a perpetrator, witness bullying, and mixed bullying experience) was also explored.

Findings of this study would broaden our understanding of bullying on college campuses and enable decision-makers as well as practitioners to develop interventions to effectively prevent or reduce bullying to create a safer educational environment for students’ learning. Moreover, it will have significant contribution to the literature of bullying on college campuses cross-culturally. Also, it will direct the focus to sustainable prevention and intervention strategies that work with the whole university by involvement of parents, instructors as well as stakeholders.

## Materials and Methods

### Study Design

A cross-sectional study was conducted on 839 students from a national university in the UAE. Ethical approval from the university’s ethical committee was obtained during the academic year 2016–2017 (REC No. ZU14_122_F). Data collection was carried out between Fall 2017 and Spring 2018.

### Sampling

The total number of students enrolled at the university at the time of data collection in both the Dubai and Abu Dhabi campuses was approximately 9,000 (4,000 in Dubai and 5,000 in Abu Dhabi) Emirati undergraduate students. To have 10% of the total population representative of all colleges, the estimated sample size was 900 students. Participants were selected using stratified random sampling (the stratum was the college name). The sampling unit was the class. The classes, student numbers, and locations of the classes were imported from the university’s Banner Web. Simple random sampling was used to obtain a list of classes from each college. The instructor of each selected class was contacted *via* e-mail to assign appointment for data collection and was informed of the study objective. Trained research assistants visited the selected classes and collected data, resulting in 839 questionnaires from both Dubai and Abu Dhabi.

### Participants

Eight hundred and thirty-nine college students from a national university in the UAE participated in this cross-sectional study. Of those, 744 (97.5%) were recruited from undergraduate programs, such as Communication and Media Sciences, Technical Innovation, Humanities, Natural Sciences and Public Health, and the first year Academic Bridge Program. Eight hundred and four (95.8%) were females and 35 (4.2%) were males. Their mean age was 20.76 years old (SD = 2.35). As for nationality, 803 (96.7%) were Emiratis and the remaining 27 (3.3%) were from other nationalities (e.g., Omani, Saudi, Yemeni, Sudanese, Palestinian, Lebanese, and American). The majority of the sample 728 (86.8%) were single, 106 (12.6%) were married, and 5 (0.6%) were either divorced or engaged. Most participants were in their third year 288 (34.3%) or fourth year of study 244 (29.1%), and the remaining were either in their first 135 (16.1%) or second year 121 (14.4%) and 51 (6.1%) did not provide data.

### Measures

#### Bullying Questionnaire

The Bullying questionnaire was designed for this study (see Appendix). The style of the questionnaire is in line with those of Campbell et al. (2012) and Tanrikulu and Campbell (2015). As definitions improve the validity of responses [Solberg and Olweus (2003); cited in Tanrikulu and Campbell, 2015], bullying in this study was defined as:

“Any repeated behavior aimed at causing harm (physical, mental, or psychological) to or for practicing control over a person. It can be physical (e.g., hitting and kicking) or verbal (e.g., name calling, gossiping, and threat) or social (e.g., destroy friendships and reputation), or cyber bullying (e.g., use of Internet to hurt a person).”

The questionnaire consisted of two parts labeled A and B. Part A comprised of eight demographic questions, such as gender, age, marital status, year in the university (first year, second year, third year…. etc), major, educational level (undergraduate or graduate), and nationality. In Part B, participants were asked “Have you been bullied in the university?.” Those who answered “Yes” were asked to proceed to answer 10 questions related to frequency of bullying, who were the aggressors, types of bullying, emotional experience after being bullied, response to bullying, reasons for being bullied… etc. For each question, respondents were given options to choose from. For example, in Question 1 “How many times have you been bullied in the University?,” respondents were asked to choose “1 time, or 2 times, or 3 times, or more than 3 times.” For Question 2: “Were you bullied by (you can choose more than 1)?,” three options were provided “A student, A group of students, Instructor/Faculty, Employee.” For Question 3 “What kind of bullying were you exposed to?,” four options were provided to choose from “(a) physical (hitting, hair pulling, kicking etc.), (b) verbal (being laughed at, bad jokes and comments, name calling, shouting at etc.), (c) on social media (got harassed through Facebook, Twitter, Instagram,….etc), (d) others (specify).” For Question 4, “How did you feel after been exposed to bullying?,” responses were (a) scared, (b) anxious, (c) depressed, (d), unable to concentrate on studying, (e) angry, and (f) other feeling (specify).

Those who answered “No” to bullying experiences were instructed to proceed directly to question 7 “In general, what are some of the reasons some students got bullied?” through 10 “How do you think the university should deal with bullying and aggressive student behavior? Give 2–3 suggestions.” In Question 7, options of reasons provided were, (a) jealousy, (b) physical appearance, (c) hate, (d) nationality, and (e) other (specify). As the Bullying Questionnaire was a checklist-response type, two psychology experts reported its face validity.

#### Aggression Questionnaire

The Aggression Questionnaire (AQ; Buss and Perry, 1992) was adopted to measure aggression behavior among college students. It consisted of 29 items measuring physical aggression, verbal aggression, anger, and hostility. Sample items are “I have become so mad that I have broken things,” “I tell my friends openly when I disagree with them,” and “I am an even-tempered person.” Items are rated on a five-piont Likert scale from 1 (extremely uncharacteristic of me) to 5 (extremely characteristic of me) with two items (i.e., 9 and 16) positively worded so that they are reversed in scoring. The scale’s developers reported four subscales, namely, Physical Aggression, Verbal Aggression, Anger, and Hostility. Total score is the sum of scores on all items which can range from 29 to 145 with higher scores meaning greater aggression. The overall scale and four subscales were found to be reliable (alphas were ranging from 0.72 to 0.85 for the subscales and 0.89 for the total scores and test–retest of 0.72 to 0.80 for the subscales and the total scores) and valid. It was found to be correlated with other personality traits, such as emotionality, self-esteem, impulsiveness, assertiveness, competitiveness, public, and private self-consciousness. Additionally, the scale discriminated between males and females with males scoring higher in all the subscales except on Anger (Buss and Perry, 1992).

The scale was translated into Arabic by AlSheikh et al. (2011) using high school samples in the UAE. AlSheikh et al. (2011) found AQ to be reliable (alphas were 0.64 to 0.80 for the subscales and 0.94 for the total scores). We found AQ to be an unidimensional scale with alpha of 0.90.

#### Posttraumatic Stress Disorder Checklist for DSM-5

The PCL-5 (Weathers et al., 2013) is a 20-item self-reported questionnaire, corresponding to the Diagnostic and Statistical Manual of Mental Disorders, 5th Edition (DSM-5) symptoms criteria for Post-Traumatic Stress Disorder (PTSD). It was selected to measure symptoms of post-traumatic stress in this research. The wording of PCL-5 items reflects both changes to existing symptoms and the addition of new symptoms in the DSM-5. Sample items include: in the past month how much you have been bothered by “repeated, disturbing, and unwanted memories of the stressful experience?,” “trouble remembering important parts of the stressful experience,” and “trouble falling or staying asleep?.” Items are rated on a four-point Likert scale, ranging from 0 (not at all) to 4 (extremely). The total scores can be obtained by adding the scores for each of the 20 items with higher scores indicating increased severity of symptoms. In two studies, using college student samples, Blevins et al. (2015) reported high internal consistency (*α* = 0.95 and 0.94) and test–retest reliability (*r* = 0.82). As for validity, correlations between 0.25 and 0.77 were obtained for the PCL-5 using measures of PTSD, personality, depression, anxiety, and other psychological problems. For example, the PCL-5 demonstrated its convergent validity (*r* = 0.74–0.85) and discriminant validity (*r* = 0.31–0.60) with measures of related (e.g., depression) and unrelated constructs (e.g., antisocial personality features and mania).

Following the guidelines of the International Test Commission (2001) for translating tests, in this study, the PCL-5 was translated into Arabic using translation–back translation method. Two professional translators translated the PCL-5 into Arabic and two bilingual psychology experts translated it back to English. Discrepancies in translation and back translation were discussed and resolved. As recommended by the developers of the checklist, nine items (i.e., 1, 2, 3, 4, 5, 6, 7, 8, and 10) were worded in reference to bullying experiences. For example, item 1 was changed to “repeated, disturbing, and unwanted memories of the bullying experience?” And item 8 was worded to “trouble remembering important parts of the bullying experience?.”

We found a 2-factor solution for the PCL-5. Alpha was found to be 0.94 and 0.93 for factor 1 (measuring depressive and anxiety symptoms) and factor 2 (assessing the cognitive aspect of the trauma), respectively, and 0.95 for the total scale.

#### The Primary Care Anxiety and Depression Scale

The PCAD (El-Rufaie et al., 1997) consists of 12 items designed to measure anxiety and depression (e.g., do you experience sudden feelings of panic?). The PCAD is rated on a four-point Likert scale, ranging from 0 (non-case) to 3 (severe), with high scores indicating high levels of anxiety and depression. El-Rufaie et al. reported a Cronbach’s alpha of 0.91 for its reliability and found the scale to be a valid instrument for detecting clinically significant anxiety and depression in Arab populations. They found PCAD to be correlated strongly with the psychiatrist’s assessment (*r* = 0.61), as compared to its correlation with the general practitioners’ assessments (*r* = 0.23). Al-Darmaki (2014) reported Cronbach’s alpha of 0.89 for groups of college student users and non-users of counseling. In the present study, Cronbach alpha was 0.81, suggesting a good internal consistency reliability.

#### Procedure

A survey including a consent form, demographic information, the bullying questionnaire, AQ, PCAD, and PCL-5 was first piloted on a sample of 35 college students who were not included in the present research analysis. Results indicated moderate to high reliabilities for the three scales used in this study. Cronbach’s alpha coefficients were 0.93 for AQ and 0.84–0.66 for its subscales and.81 and 0.80 for PCAD and PCL-5, respectively. Feedback from the pilot study was used to revise the survey before using it in this study. The survey was distributed to participants in their classes and was informed that participation is voluntary and that there was no penalty for refusing to participate. They were also informed that their data will be confidential.

Data were analyzed in three steps. First, reliabilities and validities of the three scales (AQ, PCL-5, and PCAD) were obtained through internal consistency and correlations. Second, frequencies, means, and SDs were calculated. Third, *t*-tests were obtained for the three psychological measures for each of the bullying experiences being reported (i.e., being bullied, a perpetrator, witness, or mixed bullying experience). Fourth, ANOVA tests were used.

## Results

### Bullying Experience

Results showed that 26.3% of the sample experiencing bullying. Of the 26.3, 8.7% (*n* = 72) reported being victimized (Table 1). Of those, 45.8% reported being bullied one time, 31.9% reported being bullied twice, and 22.2% reported being exposed to bullying three times.

**Table 1:** Breakdown of reported bullying experience by gender.

Bullying experience	Males (*n* = 35)	Females (*n* = 804)	Total	%
Victims	3	69	72	8.7
Perpetrators	4	25	29	3.6
Bystanders	11	174	185	22.8
Mixed bullying experience	4	54	58	6.9

*N* = 839.

Most of the victims reported that the aggressors were students (46.6%), or a group of students (43.1%), and only 8.6% reported being bullied by a faculty/instructor or a university staff (1.7%).

With regards to types of bullying, 92.2% reported being verbally bullied and 4.7% reported being bullied through social media. Only one case reported being exposed to physical bullying and another one reported experiencing property damage, “my car was damaged.” With regards to feelings after exposure to bullying, most of those who got bullied reported feeling angry (46.6%), depressed (17.2%), anxious (8.6%), or other feelings, such as feeling annoyed, uncomfortable, crying, feeling disgusted, inattentive, numbness (20.7%), or unable to concentrate on studying (5.2%), or scared (1.7%).

As for their response to bullying, 45.2% showed no reaction, 30.6% told a friend, 8.6% informed Student Affairs, 11.3% reacted with revenge and self-defense or informed a family member, 3.2% informed their advisors, and one case called the university security. As for reasons for being bullied, 52.4% thought they were bullied for jealousy, 12.7% believed they were bullied for being disliked, 11.1% reported being victimized for their physical appearance, and 22.1% believed that they were bullied for other reasons (i.e., differences in opinion, academic success, and being liked by the faculty). Only one student mentioned her nationality as the reason for being bullied.

Of those who responded to the question regarding bullying others, 3.6% reported that they were involved in bullying others as a result of being bullied. Of those, eight respondents indicated that they bullied others for their physical appearance, four students mentioned that they bullied others for retaliation, three students bullied others for hate, and six students engaged in bullying for other reasons (i.e., disagreement, disrespect of others, desire to control, to show strength, peer influence, having psychological problems, inferiority complex, and family neglect) and the remaining 8 students did not provide data.

Of the 811 students who responded to a question about witnessing bullying, (22.8%) witnessed friends being bullied. Of those bystanders, more than half (58.2%) tried to help the victims, 30.5% reported that they ignored and did not react, 6.2% mentioned that they did other things (e.g., providing support and empathy, asking the victims’ friends to ignore, joined the fight, deciding not to interact, and becoming a friend with the victim). Only 3.4% informed Student Affairs and 1.7% got scared and ran away.

Results also revealed that of those who said “Yes” to bullying, 6.9% reported experiencing more than one form of bullying (e.g., being victims, or perpetrators, or bystanders).

As for suggesting ways for the university to deal with bullying, the most frequent responses were dismissal (33%), awareness programs (32.5%), warnings (25.8%), and introducing new rules to deal with bullying on campus (19.2%).

### Descriptive Statistics and Correlations

Findings (Table 2) showed that the mean scores for the sample was 53.94 (SD = 16.47) for AQ, 11.98 (SD = 5.81) for PCAD, and 17.26 (SD = 17.22) for PCL-5. These figures suggest that the participants exhibited moderate aggressive personality traits, low levels of depression and anxiety, and PTSD symptoms. Correlation between AQ and PCAD was *r* = 0.45, indicating positive association between aggression and depression and anxiety. The correlation between Aggression and PCL-5 was *r* = 0.42 and between PCAD and PCL-5 was *r* = 0.57. These correlations were significant at *p* < 0.05 and were in the expected directions, providing additional evidence for the validity of the scales.

**Table 2:** Correlations, means, and SDs for the total sample.

Measure	1	2	3	*M*	*SD*
AQ	—	0.45^*^	0.42^*^	53.94	16.47
PCAD		—	0.57^*^	11.98	5.81
PCL-5			—	17.26	17.22
*M*	53.94	11.98	17.26		
*SD*	16.47	5.81	17.22		

AQ, aggression questionnaire; PCAD, the primary care anxiety and depression scale; and PCL-5, posttraumatic stress disorder checklist for DSM-5.

* *p* < 0.05.

### *T*-test

A series of t-tests were performed for each of the bullying behavior type (i.e., victims, perpetrators, bystanders, and mixed bullying experience) to examine if there were mean differences in their mean scores on each of the three psychological scales (i.e., AQ, PCAD, and PCL-5). Results indicated significant mean scores differences between each of the four groups of bullying experience and those who did not experience bullying on all the psychological measures.

Results are demonstrated in Tables 3 and 4. The mean scores of group 1 (victims), group 3 (bystanders) and Group 4 (those who reported mixed bullying experience) on the psychological measures (AQ, PACD, and PCL-5) were significantly different than those who did not experience bullying, *p* < 0.05. However, for group 2 (perpetrators) their mean scores on AQ only (*M* = 72.35; SD = 18.69) were significantly different from those who did not report exposure to any bullying experience (*M* = 53.29; SD = 16.10), *t* = 5.18; *df* = 630, *p* < 0.05.

**Table 3:** Means and SDs for the psychological variables for the four forms of bullying experience.

Bullying experience	AQ			PCAD			PCL-5		
	*M*	*SD*	*n*	*M*	*SD*	*n*	*M*	*SD*	*n*
*Victims*									
Yes	64.09	19.47	57	14.70	6.61	67	27.09	19.29	66
No	52.93	19.89	588	11.71	5.64	731	16.33	16.73	666
*Perpetrators*									
Yes	72.35	18.69	20	13.44	6.33	27	23.92	20.12	25
No	53.29	16.10	612	11.90	5.81	749	16.82	17.03	686
*Bystanders*									
Yes	61.05	16.57	140	13.93	5.97	176	23.61	16.87	160
No	52.13	15.96	494	11.41	5.68	603	15.37	16.99	555
*Mixed bullying*									
Yes	68.12	19.16	42	15.06	6.89	54	29.04	18.66	51
No	51.45	15.58	483	11.35	5.61	597	15.12	16.67	546

AQ, aggression questionnaire; PCAD, the primary care anxiety and depression scale; and PCL-5, posttraumatic stress disorder checklist for DSM-5.

**Table 4:** Mean differences between the groups on the psychological variables.

Group/Variables	*t*	*Df*	Sig
*Group 1*			
AQ	4.19^*^	63.44	0.000
PCAD	4.09^*^	75.09	0.001
PCL-5	4.37^*^	75.00	0.000
*Group 2*			
AQ	5.18^*^	630	0.000
PCAD	1.35	774	0.177
PCL-5	1.74	25.27	0.094
*Group 3*			
AQ	5.79^*^	632	0.000
PCAD	5.13^*^	777	0.000
PCL-5	5.41^*^	713	0.000
*Group 4*			
AQ	5.49^*^	45.84	0.000
PCAD	3.83^*^	59.52	0.000
PCL-5	5.66^*^	595	0.000

Group 1, victims; Group 2, perpetrators; Group 3, bystanders; Group 4, mixed bullying (any combination of groups 1–3). *N* for each group is based on the number of participants who provided complete answers for each of the scales. AQ, aggression questionnaire; PCAD, the primary care for anxiety and depression scale; and PCL-5, posttraumatic stress disorder checklist for DSM-5.

* *p* < 0.05.

### Analysis of Variance

Based on the Bullying Questionnaire, the four groups of participants were created and only those who provided complete data were included in the analysis. For each of the psychological measures and based on responses from participants, the mean scores and SDs as well as the number of victims, perpetrators, bystanders, and those who reported mixed bullying experience are shown in Table 5. Table 6 showed that the between groups one-way ANOVA was significant (*p* < 0.05) for the four groups who experienced bullying, in particular, there was a between groups significant difference (*p* < 0.05) on AQ [*F* (4,647) = 14.52], *p* = 0.000. For PCAD, there was a between groups significant differences at *p* < 0.05 [*F* (4,801) = 7.82], *p* = 0.000. Similarly, for PCL-5, there was a between groups significant differences at *p* < 0.05 [*F* (4,735) = 10.96], *p* = 0.000.

**Table 5:** Descriptive statistics for the psychological measures for participants exposed to bullying experience and for those who were not exposed to bullying.

Scale/Bullying experience	*N*	*M*	*SD*
*AQ*			
Uninvolved	483	51.45	15.69
Victims	19	56.79	18.24
Perpetrators	7	62.71	15.51
Bystanders	101	58.82	15.00
Mixed bullying experience	42	68.12	19.16
Total	652	53.94	16.47
*PCAD*			
Uninvolved	597	11.35	5.61
Victims	21	13.05	6.29
Perpetrators	9	12.67	6.75
Bystanders	125	13.38	5.43
Mixed bullying experience	54	15.06	6.89
Total	806	11.98	5.81
*PTSD*			
Uninvolved	546	15.12	16.67
Victims	22	24.18	20.63
Perpetrators	8	15.50	18.09
Bystanders	113	21.10	15.45
Mixed bullying experience	51	29.04	18.66
Total	740	17.26	17.22

AQ, aggression questionnaire; PCAD, the primary care for anxiety and depression; and PCL-5, posttraumatic stress disorder checklist for DSM-5.

**Table 6:** One-way between groups ANOVA on the variables under investigation.

Measure	Mean square	*df*	*F*	Sig
*AQ*				
Between groups	3637.61	4	14.52^*^	0.000
Within groups	250.51	647		
Total		651		
*PCAD*				
Between groups	254.93	4	7.82^*^	0.000
Within groups	32.59	801		
Total		805		
*PTSD*				
Between groups	3082.57	4	10.96^*^	0.000
Within groups	281.33	735		
Total		739		

AQ, aggression questionnaire; PCAD, the primary care for anxiety and depression; and PCL-5, posttraumatic stress disorder checklist for DSM-5.

* *p* < 0.05.

## Discussion

### Bullying Prevalence, Typology, and Gender Considerations

In comparison with other studies (e.g., Kraft and Wang, 2010; Lindsay and Krysik, 2012; Rospenda et al., 2013; Sobba et al., 2017; AlMulhim et al., 2018) our findings showed low rates of bullying among national university students in the UAE. This prevalence rate is in line with a previous study that found UAE to have the lowest rate of bullying among a sample of middle-school students from 19 countries (Fleming and Jacobsen, 2010). This is also in line with international studies on college students which reported lower levels of bullying behavior (Beran et al., 2012; Bauman and Newman, 2013; Schenk et al., 2013; Chen and Huang, 2015; Giovazolias and Malikiosi-Loizos, 2015). Nevertheless, the differences in bullying rates reported in the literature may be partly explained by the use of different types of measures; bullying definition, student perceptions of bullying behaviors, cultural norms, and specific personal student characteristics. Future research should focus on establishing a clear standard definition of bullying and different types of bullying also needs to be established according to United Nations Education and Scientific Cultural Organization (2019) to allow for accurate comparisons across different cultures.

Our results showed that most bullying occurs using traditional ways (face-to-face bullying, verbal, physical…etc.) and only a small percentage of bullying occurs using social media/cyberbullying. Traditional face-to-face bullying, especially verbal aggression and relational/indirect bullying (e.g., spreading rumors and being excluded) are still the most common types of bullying (Lund and Ross, 2017). A decrease in direct physical aggression compared to an increase in incidents of indirect bullying is typically found in the literature, possibly due to the development of verbal and social skills with age (Pepler et al., 2008; Craig et al., 2009).

Consistent with previous research, our study showed that most victims who reported being bullied were females. Although males are more likely to bully others and get bullied (Napolitano, 2011), females are more likely to report being bullied. Physical aggression and direct bullying has been regularly associated with males, whereas relational aggression has mostly been associated with males (Hinduja and Patchin, 2008; Olweus and Limber, 2010). In our study, however, it was impossible to detect any meaningful gender differences in the rate of bullying due to the small number of male participants. Future research should consider using a balanced male–female sample size to allow for gender comparison taking into consideration variables, such as types of bullying, school response to bullying, and the contributions of various risk factors (e.g., physical appearance, nationality, socio-economic status, disability, and race).

### Role of Friends, Families, and Bystanders in Bullying

Respondents were mostly reluctant to report bullying incidents to university officials as only a small percentage reported the bullying incidents to the concerned university staff. Student reluctance to report bullying may be due to embarrassment and perceived negative outcomes (Juvonen and Gross, 2008; Boulton et al., 2017). Some victims, however, were able to tell a friend. This is not surprising as friends have been identified as a source of help for problems experienced by college students in the UAE (Al-Darmaki, 2011). This result is in line with previous research that found friendship to be a protective factor against victimization (Burns et al., 2010; Méndez et al., 2017).

Although families play a critical role in providing emotional support, encouraging their children to disclose bullying incidents, and teaching them coping skills (Al-Darmaki, 2011; Abdirahman et al., 2013; Johnson et al., 2013), the role of parents in the bullying behavior of their children was not investigated in this study. Future studies should examine the role of parents in supporting or preventing bullying behavior of their children (Hinduja and Patchin, 2008; Tanrikulu and Campbell, 2015).

Respondents in the present study reported being mostly bullied by peer students or by a “group” of students. This is an illustration of the bullying circle or group process, where some of the bystanders are likely to have joined the bullying by taking the role of “assistants/henchmen” and “reinforcer.” Self-enhancement and self-protective motives are likely to encourage bystanders to join the bullying (Juvonen and Gross, 2008). Also, culturally, it is expected in the UAE to conform in order to be accepted by peers.

When examining bystander behavior further, many respondents witnessed friends being bullied. More than half of the witness bystanders (that are not assistants or reinforcers of the bullying) tried to support their friends during the bullying episode, thus taking on the role of defending, while others helped by reporting the aggressors or comforting the victim. Such defending characteristics are associated with having high self-empathy, self-efficacy, and social status, which drives witnesses to intervene (Pozzoli and Gini, 2010). The remainder of the bystanders did nothing, and remained silent, while a small number of witnesses admitted being afraid and running away from the situation. This is a typical pattern in the literature, where it is rare for bystanders to help or defend the victim and they are usually passive onlookers (Pozzoli and Gini, 2010).

Most of the participants did not bully others as a result of being bullied. This result implies that respondents who were previously bullied may have higher empathy and sensitivity toward others, as well as self-efficacy/regulation and moral engagement and resilience (Sapouna and Wolke, 2013; Holt et al., 2014).

Faculty/staff bullying of students were much lower in comparison to the higher levels found in the literature (Al-Hussain et al., 2008; Marraccini et al., 2015). Some of these studied reported 18% bullying by university/college faculty (Chapell et al., 2004) to 44% bullying cases by teachers in schools (Marraccini et al., 2015). The reasons behind faculty bullying of students is worth investigating as there seems to be a range of factors (e.g., being burned out or envious of smarter students) that are attributing to teacher-bullying behavior (Twemlow et al., 2006). Faculty/staff who received training and participated in a bullying prevention program felt more confident in dealing with bullying situations, had more supportive attitudes toward victims of bullying, and felt more positive about collaborating with parents regarding bullying problems (Alsaker, 2004; Carissa Fehr and Seibel, 2022).

### Bullying and Mental Health

Victims in this research reported experiencing negative psychological impacts after being exposed to bullying. They reported more externalized symptoms, such as feelings of anger, discomfort, disgust, numbness, crying, and inability to concentrate on their education. Furthermore, they experienced more internalized symptoms, such as depression, anxiety, and fear. This is in line with the large body of literature that found exposure to bullying to have a serious impact on the wellbeing of youth (e.g., Beran et al., 2012; Tanrikulu and Campbell, 2015; Ranney et al., 2016). These may last throughout their lives (Williams and Guerra, 2007; Copeland et al., 2013; Takizawa et al., 2014). Also, this is in line with Fleming and Jacobsen (2010) findings that bullying among students from the UAE was associated with sadness, hopelessness, loneliness, insomnia, and suicide thoughts.

Results based on the psychological measures revealed a moderate level of aggressive personality traits and low levels of symptoms of depression, anxiety, and PTSD. These findings show that those who have experienced bullying had significantly higher levels of depression and anxiety, and PTSD symptoms compared with those who had no bullying experience. These results are also supported by previous findings (Beran et al., 2012; Landstedt and Persson, 2014; Ranney et al., 2016; AlMulhim et al., 2018) that bullying has a negative impact on college students’ psychological wellbeing.

As students tend to be reluctant to seek counseling (Al-Darmaki, 2011, 2014), the university student counseling centers should plan outreach programs targeting the victims, the aggressors, and bystanders to increase their awareness of the negative impact of bullying on their wellbeing. As cases of bullying may not be reported for fear of retaliation from peers, student community should be encouraged to report incidents to counselors so that care can be provided to those who are involved in bullying. Also, investigating the impact of bullying on students’ academic performance (Hinduja and Patchin, 2008) in future studies may reveal interesting results.

Those who bullied others exhibited significantly higher levels of aggression as compared with those who did not experience any form of bullying. This suggests that they possess more aggressive traits than their peers which may explain in part, their tendency to bully others. This is consistent with previous research (e.g., Undheim and Sund, 2010; Sigurdson et al., 2014).

### Bullying Preventions

Although participants’ most frequent recommended strategies for dealing with bullying was dismissal of the aggressors, if they ignore a written warning after the first episode, such action would have negative implications for students and their parents. Suspending students for problematic behavior may place them at a higher risk of academic failure, alienation, future antisocial behavior, or other social problems (American Academy of Pediatrics, 2003; Arcia, 2006). The most effective bullying prevention programs are whole school approaches in combination with a multi-tiered public health model (for reviews see Rivara and Le Menestrel, 2016). Anti-bullying programs are usually most efficient when implemented with older students rather than younger ones (Smith et al., 2003).

### Limitations

Despite its interesting findings, this study has some limitations. The definition provided for bullying in the survey may not have been broad enough to capture all types and forms of bullying. Participants may have relied on their own understanding of bullying to report their experiences. Therefore, they may have reported what they perceived as bullying which means that some forms of bullying may have not been considered (United Nations Education and Scientific Cultural Organization, 2009; AlMulhim et al., 2018). Future research should provide explicit definitions of the full range of bullying behaviors to help identify them more accurately. Also, poly-victimization was not controlled (Ford and Delker, 2018). Researchers have discussed the limitations of the cross-sectional approach (e.g., Landstedt and Persson, 2014), with no mechanism to establish a temporal relationship, this study was unable to determine whether bullying leads to mental health problems and aggression or if these factors pre-date bullying. Another limitation is the use of a self-report screening measure for posttraumatic stress symptomatology, not a diagnostic interview for PTSD (Blevins et al., 2015). The incidence of PTSD may therefore be over- or under-reported. Although some participants reported inability to concentrate on their studies, the impact of bullying on academic performance was not examined. In this study, the psychological variables (i.e., aggression, depression and anxiety, and PTSD) have been measured using Likert scale and analyzed using parametric tests which might be not the best appropriate choice. Additionally, two of the reported correlations are below 0.50 and, therefore, caution should be practiced in interpreting these results. Lastly, the current study was based on a sample that consisted predominantly of Emirati university students and, therefore, its generalizability to other settings might be limited.

In conclusion, this study provides a unique contribution to our understanding of bullying behavior of college students within UAE. As systematic reporting of incidences of bullying (United Nations Education and Scientific Cultural Organization, 2019) is important to our understanding of this behavior. The findings of the present study can serve as a baseline for future research in this area. Our findings showed that incidents of bullying exist in the university setting and have adverse impact on students’ mental health. The need for frequent data collection to discover trends (increase or decrease in bullying behavior) among college student population is also crucial (United Nations Education and Scientific Cultural Organization, 2019).

## Conclusion

Experiences of bullying seem to impact college students’ mental health in the UAE. Most bullying occurs using traditional ways, such as face-to-face bullying, verbal, and physical. Only a small percentage of bullying occurs using social media/cyberbullying. The lower rates of cyberbullying than traditional bullying could be due to cyberbullying being a recent type of bullying. Many respondents witnessed friends being bullied. More than half of the witness bystanders tried to support their friends during the bullying episode. This is expected, as Emirati cultural norms are characterized with strong sense of moral obligation toward fellowship, helping others, and rescuing those in needs. Most of the participants did not bully others as a result of being bullied. Empirical research is needed to investigate this issue further. Faculty/staff bullying of students were much lower in comparison to the higher levels found in other studies. In the UAE, faculty have a highly influential role in developing and nurturing students. Therefore, it is important to raise their awareness of bullying to reduce, detect and deal with bullying effectively. Strategies for dealing with bullying and Promoting students’ wellbeing among faculty and staff can also increase their awareness of the impact of bullying on students’ mental health.

Victims reported experiencing negative psychological impacts after being exposed to bullying, such as feelings of anger, discomfort, crying, inability to concentrate on education, depression, anxiety, and fear. Those who have experienced bullying had significantly higher levels of depression and anxiety, and PTSD symptoms compared with those who had no bullying experience. There is a pressing need for psychological help for those who were exposed to bullying. Whether they had sought help from the university counseling services was not investigated. Students impacted by bullying should be encouraged to seek counseling for psychological support. Students should be encouraged to report incidents to counselors so that care can be provided to those who are involved in bullying.

There is a need to investigate the impact of bullying on students’ academic performance. Those who bullied others exhibited significantly higher levels of aggression as compared with those who did not experience any form of bullying. Although jealousy and physical appearance seem to be among the main reasons for bullying in our study, future research needs to investigate in depth reasons for bullying to broaden our understanding of such factors so that strategies can be developed to tackle this issue.

It is important to focus on sustainable prevention and intervention strategies that work with the entire university by involving staff and faculty as well as other stakeholders, such as parents. Effective bullying prevention programs are whole school approaches in combination with a multi-tiered public health model.

Therefore, efforts need to focus on developing preventive programs to increase students’ awareness of bullying and its negative impact on campus environment. Offering psychological help for those who were exposed to bullying would help them to deal effectively with this trauma.

## Data Availability Statement

The original contributions presented in the study are included in the article/Supplementary Material; further inquiries can be directed to the corresponding author.

## Ethics Statement

The studies involving human participants were reviewed and approved by Research Ethics Committee (REC)-Zayed University, Abu Dhabi, UAE. The approved committee’s reference number is ZU14_122_F. We have used a written informed consent which was given to all participants. The patients/participants provided their written informed consent to participate in this study.

## Author Contributions

DH contributed in the research initial design and the first draft of the manuscript. All authors contributed to the article and approved the submitted version.

## Funding

Zayed University funded this research (grant numbers R16066, 2016) and approved the application to conduct this research (ZU14_122_F).

## Conflict of Interest

The authors declare that the research was conducted in the absence of any commercial or financial relationships that could be construed as a potential conflict of interest.

## Publisher’s Note

All claims expressed in this article are solely those of the authors and do not necessarily represent those of their affiliated organizations, or those of the publisher, the editors and the reviewers. Any product that may be evaluated in this article, or claim that may be made by its manufacturer, is not guaranteed or endorsed by the publisher.
