# Supplementary material for: Prevalence of Bullying Behaviors Among Students From a National University in the United Arab Emirates: A Cross-Sectional Study
Source: Front Psychol. 2022 Apr 25;13:768305. doi: 10.3389/fpsyg.2022.768305 (PMC9082408; doi:10.3389/fpsyg.2022.768305)
Supplement: Supplementary file 1 [file Data_Sheet_1.PDF]

Pearson's correlation

Introduction

Often several quantitative variables are measured on each member of a sample. If we consider a pair of such variables, it is frequently of interest to establish if there is a relationship between the two; i.e. to see if they are *correlated*.

We can categorise the type of correlation by considering as one variable increases what happens to the other variable:

- *Positive correlation* – the other variable has a tendency to also increase;
- *Negative correlation* – the other variable has a tendency to decrease;
- *No correlatio*n – the other variable does not tend to either increase or decrease.

The starting point of any such analysis should thus be the construction and subsequent examination of a *scatterplot*. Examples of negative, no and positive correlation are as follows.

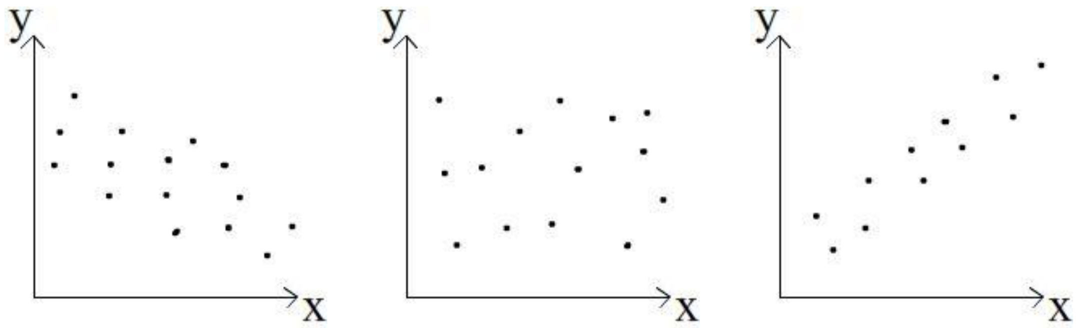

Negative correlation **No correlation** **Positive correlation**

Example

Let us now consider a specific example. The following data concerns the blood haemoglobin (Hb) levels and packed cell volumes (PCV) of 14 female blood bank donors. It is of interest to know if there is a relationship between the two variables Hb and PCV when considered in the female population.

Hb	PCV
15.5	0.450
13.6	0.420
13.5	0.440
13.0	0.395
13.3	0.395
12.4	0.370
11.1	0.390
13.1	0.400
16.1	0.445
16.4	0.470
13.4	0.390
13.2	0.400
14.3	0.420
16.1	0.450

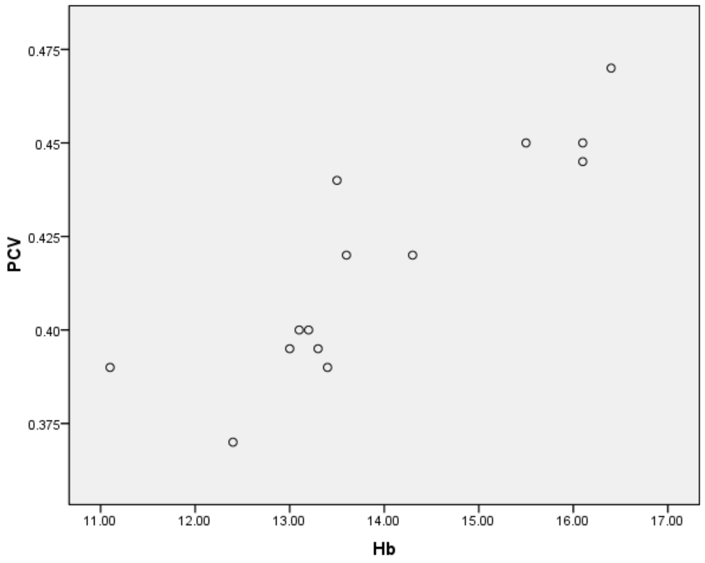

The scatterplot suggests a definite relationship between PVC and Hb, with larger values of Hb tending to be associated with larger values of PCV.

There appears to be a positive correlation between the two variables.

We also note that there appears to be a *linear* relationship between the two variables.

Correlation coefficient

Pearson's correlation coefficient is a statistical measure of the strength of a *linear* relationship between paired data. In a sample it is denoted by r and is by design constrained as follows

$$-1 \leq r \leq 1$$

Furthermore:

- Positive values denote positive linear correlation;
- Negative values denote negative linear correlation;
- A value of 0 denotes no linear correlation;
- The closer the value is to 1 or –1, the stronger the linear correlation.

In the figures various samples and their corresponding sample correlation coefficient values are presented. The first three represent the "extreme" correlation values of -1, 0 and 1:

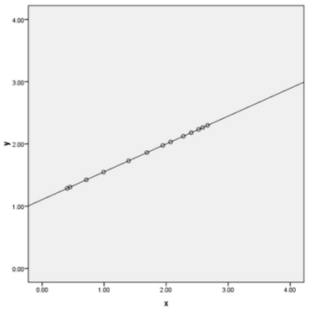

$r = -1$
perfect -ve correlation

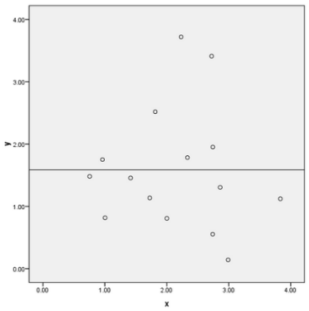

$r = 0$
no correlation

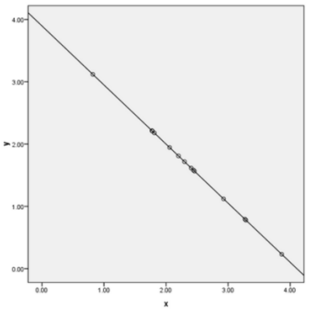

$r = 1$
perfect +ve correlation

When $r = \pm 1$ we say we have *perfect* correlation with the points being in a perfect straight line.

Invariably what we observe in a sample are values as follows:

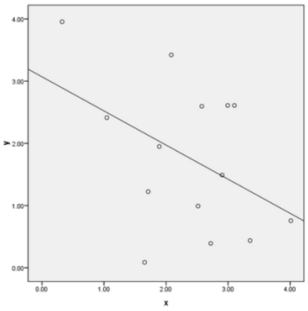

$r = -.45$
moderate -ve correlation

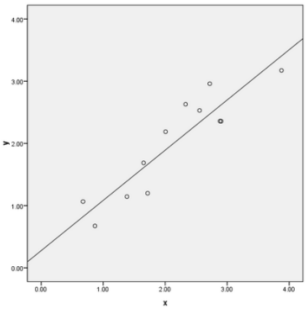

$r = .92$
very strong +ve correlation

Note:

1) the correlation coefficient does not relate to the gradient beyond sharing its +ve or –ve sign!
2) The correlation coefficient is a measure of linear relationship and thus a value of $r = 0$ does not imply there is no relationship between the variables. For example in the following scatterplot $r = 0$ which implies no (linear) correlation however there is a perfect quadratic relationship:

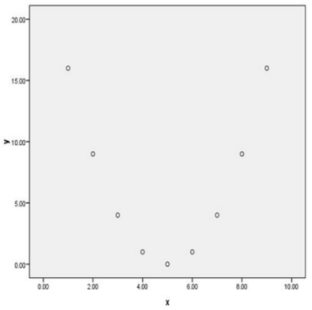

$r = 0$
perfect quadratic relationship

Correlation is an effect size and so we can verbally describe the strength of the correlation using the guide that Evans (1996) suggests for the absolute value of *r*:

- .00-.19 "very weak"
- .20-.39 "weak"
- .40-.59 "moderate"
- .60-.79 "strong"
- .80-1.0 "very strong"

For example a correlation value of $r = .42$ would be a "moderate positive correlation".

Assumptions

The calculation of Pearson's correlation coefficient and subsequent significance testing of it requires the following data assumptions to hold:

- interval or ratio level;
- linearly related;
- bivariate normally distributed.

In practice the last assumption is checked by requiring both variables to be individually normally distributed (which is a by-product consequence of bivariate normality). Pragmatically Pearson's correlation coefficient is sensitive to skewed distributions and outliers, thus if we do not have these conditions we are content.

If your data does not meet the above assumptions then use Spearman's rank correlation!

Example (revisited)

We have no concerns over the first two data assumptions, but we need to check the normality of our variables. One simple way of doing is to examine boxplots of the data. These are given below.

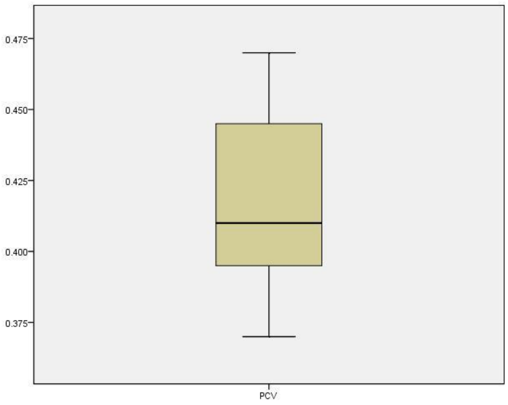

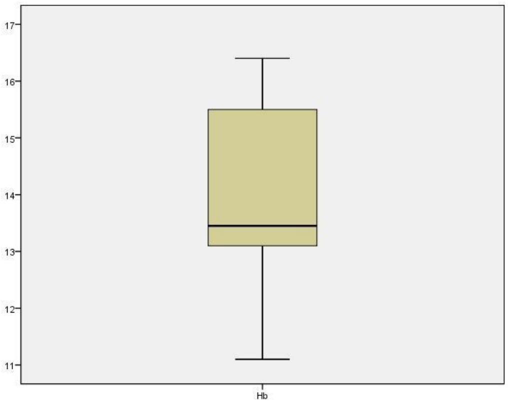

The boxplot for PCV is fairly consistent with one from a normal distribution; the median is fairly close to the centre of the box and the whiskers are of approximate equal length.

The boxplot for Hb is slightly disturbing in that the median is close to the lower quartile which would be suggesting positive skewness. Although countering this is the argument that with positively skewed data the lower whisker should be shorter than the upper whisker; this is not the case here.

Since we have some doubts over normality, we shall examine the skewness coefficients to see if they suggest whether either of the variables is skewed.

Descriptive Statistics

	N	Skewness	
	Statistic	Statistic	Std. Error
Hb	14	.262	.597
PCV	14	.299	.597
Valid N (listwise)	14		

Both have skewness coefficients that are indeed positive, but a quick check to see if these are not sufficiently large to warrant concern is to see if the absolute values of the skewness coefficients are less than two times their standard errors. In both cases they are which is consistent with the data being normal. Hence we do not have any concerns over the normality of our data and can continue with the correlation analysis.

For the Haemoglobin/PCV data, SPSS produces the following correlation output:

Correlations

		Hb	PCV
Hb	Pearson Correlation	1	.877**
	Sig. (2-tailed)		.000
	N	14	14
PCV	Pearson Correlation	.877**	1
	Sig. (2-tailed)	.000	
	N	14	14

**. Correlation is significant at the 0.01 level (2-tailed).

The Pearson correlation coefficient value of 0.877 confirms what was apparent from the graph, i.e. there appears to be a positive correlation between the two variables.

However, we need to perform a significance test to decide whether based upon this sample there is any or no evidence to suggest that linear correlation is present in the population.

To do this we test the null hypothesis, H_0, that there is no correlation in the population against the alternative hypothesis, H_1, that there is correlation; our data will indicate which of these opposing hypotheses is most likely to be true. We can thus express this test as:

$$H_0 : \rho = 0$$
$$H_1 : \rho \neq 0$$

i.e. the null hypothesis of no linear correlation present in population against the alternative that there is linear correlation present.

SPSS reports the p-value for this test as being .000 and thus we can say that we have very strong evidence to believe H_1, i.e. we have some evidence to believe that Hb and PCV are linearly correlated in the female population.

The significant Pearson correlation coefficient value of 0.877 confirms what was apparent from the graph; there appears to be a very strong positive correlation between the two variables. Thus large values of Hb are associated with large PCV values.

This could be formally reported as follows:

"A Pearson's correlation was run to determine the relationship between 14 females' Hb and PCV values. There was a very strong, positive correlation between Hb and PCV ($r = .88$, $N=14$, $p < .001$)."

Caution

The existence of a strong correlation does not imply a causal link between the variables. For example we can not imply that Hb causes PCV or vice versa.

Also you should be aware of the possibility of hidden or intervening variables. For instance suppose we consider the relationship between reading ability and foot length for children. A scatter plot and correlation analysis of the data indicates that there is a very strong correlation between reading ability and foot length (r = .88, N=54, p =.003):

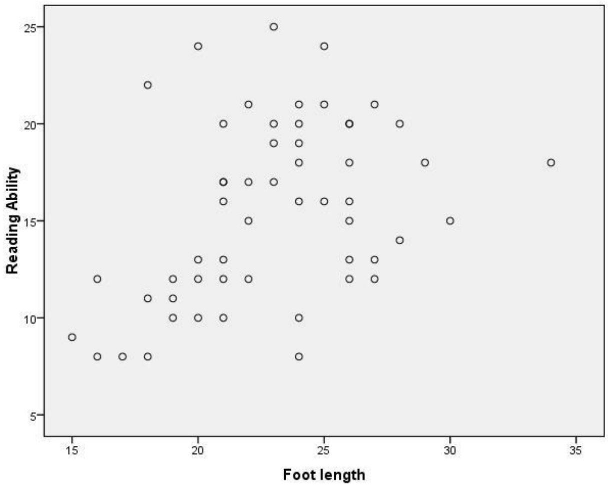

However, if we consider taking into account the children's age, we can see that this apparent correlation may be spurious.

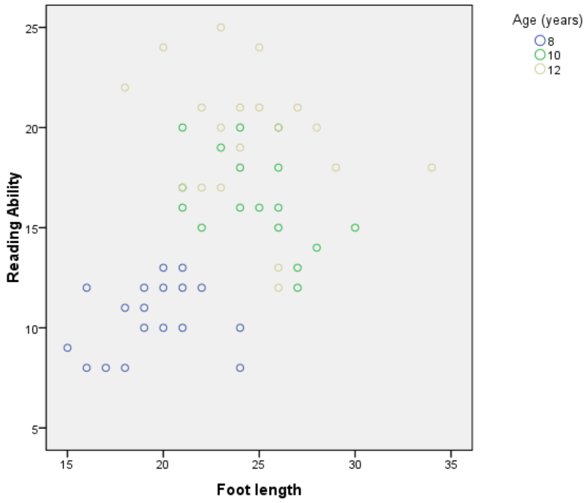

If we now reanalyse the data by age group we indeed find that in each case there appears to be no correlation between the two variables:

Age (years) = 8

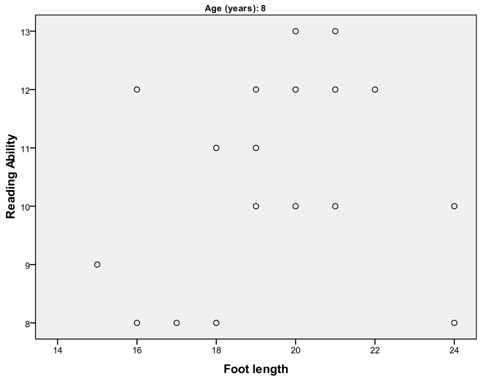

Correlations[a]

		Reading Ability	Foot length
Reading Ability	Pearson Correlation	1	.210
	Sig. (2-tailed)		.403
	N	18	18
Foot length	Pearson Correlation	.210	1
	Sig. (2-tailed)	.403	
	N	18	18

a. Age (years) = 8

Age (years) = 10

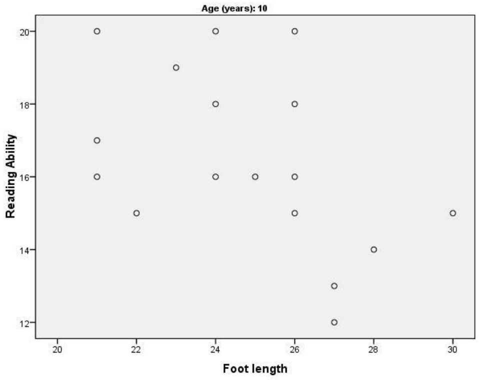

Correlations[a]

		Reading Ability	Foot length
Reading Ability	Pearson Correlation	1	-.465
	Sig. (2-tailed)		.060
	N	17	17
Foot length	Pearson Correlation	-.465	1
	Sig. (2-tailed)	.060	
	N	17	17

a. Age (years) = 10

Age (years) = 12

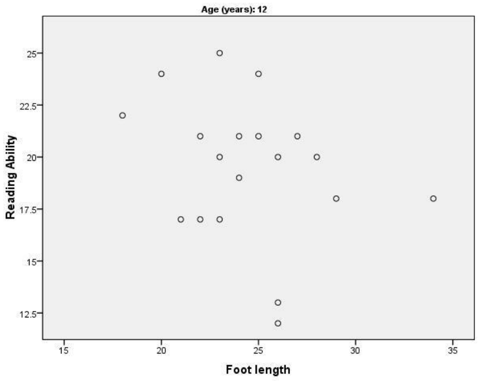

Correlations[a]

		Reading Ability	Foot length
Reading Ability	Pearson Correlation	1	-.290
	Sig. (2-tailed)		.228
	N	19	19
Foot length	Pearson Correlation	-.290	1
	Sig. (2-tailed)	.228	
	N	19	19

a. Age (years) = 12
